# Molecular metabarcoding reveals an omnivorous diet in *Smaragdinella* haminoeid snails and sheds light on their unique evolution

**DOI:** 10.1038/s41598-026-57881-9

**Published:** 2026-07-09

**Authors:** Monisha Bharate-Grevskott, Jon T. Hestetun, Ida H. Steen, Manuel António E. Malaquias

**Affiliations:** 1 Department of Natural History, University Museum, University of Bergen, Bergen, Norway; 2 NORCE Climate and Environment, Bergen, Norway; 3 Department of Biological Sciences, University of Bergen, Bergen, Norway; 4 Institute of Marine Sciences - OKEANOS, University of the Azores, Horta, 9900-140 Portugal

**Keywords:** Cephalaspidea, Ecological speciation, Gut microbiota, Omnivory, Ecology, Ecology, Evolution, Microbiology, Zoology

## Abstract

*Smaragdinella* marine snails are the only members of the family Haminoeidae and the order Cephalaspidea that inhabit hard substrates in the upper tidal zone, making them of special evolutionary interest. To investigate whether possible novel trophic adaptations coupled with unique morphological traits may underlie this ecological shift, we analysed the gut contents and microbiota of *Smaragdinella viridis* using DNA metabarcoding (COI and 16S rRNA) and scanning electron microscopy (SEM). COI metabarcoding revealed a diverse assemblage of dietary components dominated by diatoms, rotifers, small arthropods, and fungi. SEM observations partially corroborated these findings, indicating an omnivorous feeding strategy rather than strict herbivory. The gut bacterial community was dominated by Firmicutes (*Mycoplasma*), Proteobacteria (*Vibrio*, *Photobacterium*), and Fusobacteriota (*Psychrilyobacter*, *Propionigenium*), taxa associated with the degradation of complex carbohydrates and proteins. Morphological traits in *Smaragdinella*, including an increased number of gizzard plate ridges, a flattened ovoid shell, and an enlarged foot, most likely facilitate the processing of diverse food items and survival in wave-exposed environments. In addition, the functionality of the gut microbiome may contribute to dietary flexibility enhancing survival in dynamic, resource-variable tidal habitats. The adaptation of these snails to tidal hard bottom habitats could have been prompted by the acquisition of novel morphological features and by a diet shift, but the data do not permit to establish a causal relationship and alternative hypotheses may have to be considered.

**Supplementary Information:**

The online version contains supplementary material available at https://doi.org/10.1038/s41598-026-57881-9.

## Introduction

Malaquias et al.^1^ showed that herbivory represents the plesiomorphic condition among the order Cephalaspidea, with carnivory having arisen independently two or three times, suggesting that dietary specialization played a major role in the adaptive radiation of these snails.

Among the Cephalaspidea, the family Haminoeidae is the most diverse with 18 genera and 139 recognized valid species^2^ exclusively herbivorous feeding primarily on diatoms^3–7^ see Table 1 for a review). In general, dietary data for haminoeid snails are based on sporadic or opportunistic observations of gut contents resulting from scanning electron microscopy observations^5,7–15^. Yet, the diet reported for some genera like *Diniatys*, *Papawera*, and *Liloa* are only extrapolated from field observations^4,16–18^.

To date, only one study has qualitatively and quantitatively analysed the diet of a haminoeid species (^3^ for *Haminoea orbignyana* in Portugal). Malaquias et al.^3^, in addition to diatoms, found foraminifera in the gut of *Haminoea orbignyana* but forming only 0.04% of the diet composition. The latter authors have suggested that ingestion of animal remains and foraminifera, as also reported by Vayssière^19^ and Berrill^20^, is the result of accidental consumption during foraging.

The genus *Smaragdinella* A. Adams, 1848^21^ exhibits distinctive ecological traits among Haminoeidae and even among Cephalaspidea. It is exclusively intertidal and inhabits hard substrates such as exposed rocky and cobble shores, as well as overhanging branches of kiawe mesquite trees along the shoreline, whereas all other genera are mostly subtidal and dwell in soft sediments, algae, or seagrass. The unusual habitat preference of *Smaragdinella* makes the genus particularly interesting from an evolutionary point of view^22–28^. These snails are restricted to tropical and subtropical regions of the Indo-West Pacific realm, ranging from Easter Island to KwaZulu-Natal, South Africa, including Lord Howe Island off eastern Australia, extending northward to Tokyo Bay, Japan^22,29–32^.

Despite its unique ecological adaptation, the drivers behind the diversification and habitat selection of *Smaragdinella* are not understood. These snails are the only hamineoids that have a depressed body and broad muscular foot, which likely increase fitness in terms of adherence to the substrate in a hydrodynamic habitat prone to wave splashing and air exposure^28^. Another unique feature of *Smaragdinella* is the presence of a much higher number of gizzard plate ridges in the digestive tract compared with other haminoeids that may facilitate the mechanical shredding of food items^28^ permitting a more complex and diversified dietary regime. Yet the only study specifically examining the diet of *Smaragdinella* of an unidentified species from Vietnam found a predominance of diatoms^33^. However, this study was based on the analysis of fatty acids and several groups of organisms, including crustaceans, do not produce distinctive fatty acids signatures, and therefore cannot be reliably detected using this method^34^.

DNA metabarcoding is an efficient and cost-effective method for identifying dietary components, capable of detecting a wide range of taxa from gut or faecal samples^35^. This method utilizes high-throughput sequencing of taxonomically informative genetic markers, such as the mitochondrial cytochrome *c* oxidase subunit I (COI) or ribosomal DNA including the eukaryote 18S rRNA (18S) and prokaryote 16S rRNA (16S) genes, to identify prey items^36–39^. This approach has been successfully applied to a wide range of marine invertebrates such as the sea cucumbers *Stichopus monotuberculatus*, *S. chloronotus*, and *Holothuria atra*^40^, the shrimp *Pandalus borealis*^37^, the amphipods *Anonyx sarsi*, *Orchomenella minuta*, and *Gammarus* sp.^41^, the bivalve *Crassostrea gigas*^42^, and the gastropod *Littorina saxatilis*^43^. In the latter case, DNA metabarcoding revealed a broader diet than previously known, extending beyond diatoms and cyanobacteria and including the red encrusting alga *Hildenbrandia*, ciliate and flagellate protists, small invertebrates and fungi^43^.

However, metabarcoding also presents limitations. PCR amplification biases can skew the relative abundance of sequence reads, leading to under- or overrepresentation of certain taxa^44,45^. Additionally, incomplete reference databases can restrict taxonomic identification to higher levels only^43^, and contamination from environmental sources may result in false positives^46^. Despite these challenges, DNA metabarcoding significantly enhances our capacity to investigate dietary composition and ecological interactions, especially in small invertebrates for which traditional gut content analysis is difficult.

This study presents the first application of DNA metabarcoding to investigate trophic ecology in Cephalaspidea gastropods. A central question is whether the habitat shift and unique configuration of the digestive tract in *Smaragdinella* may relate to the exploitation of a distinct range of food resources, potentially increasing resilience for living on harsher tidal hard-bottom habitats. We want to test the hypothesis whether the habitat shift undergone by *Smaragdinella* was paralleled by a corresponding shift in dietary regime.

## Results

### Scanning electron microscopy of gut and faeces food contents

**Fig. 1:**
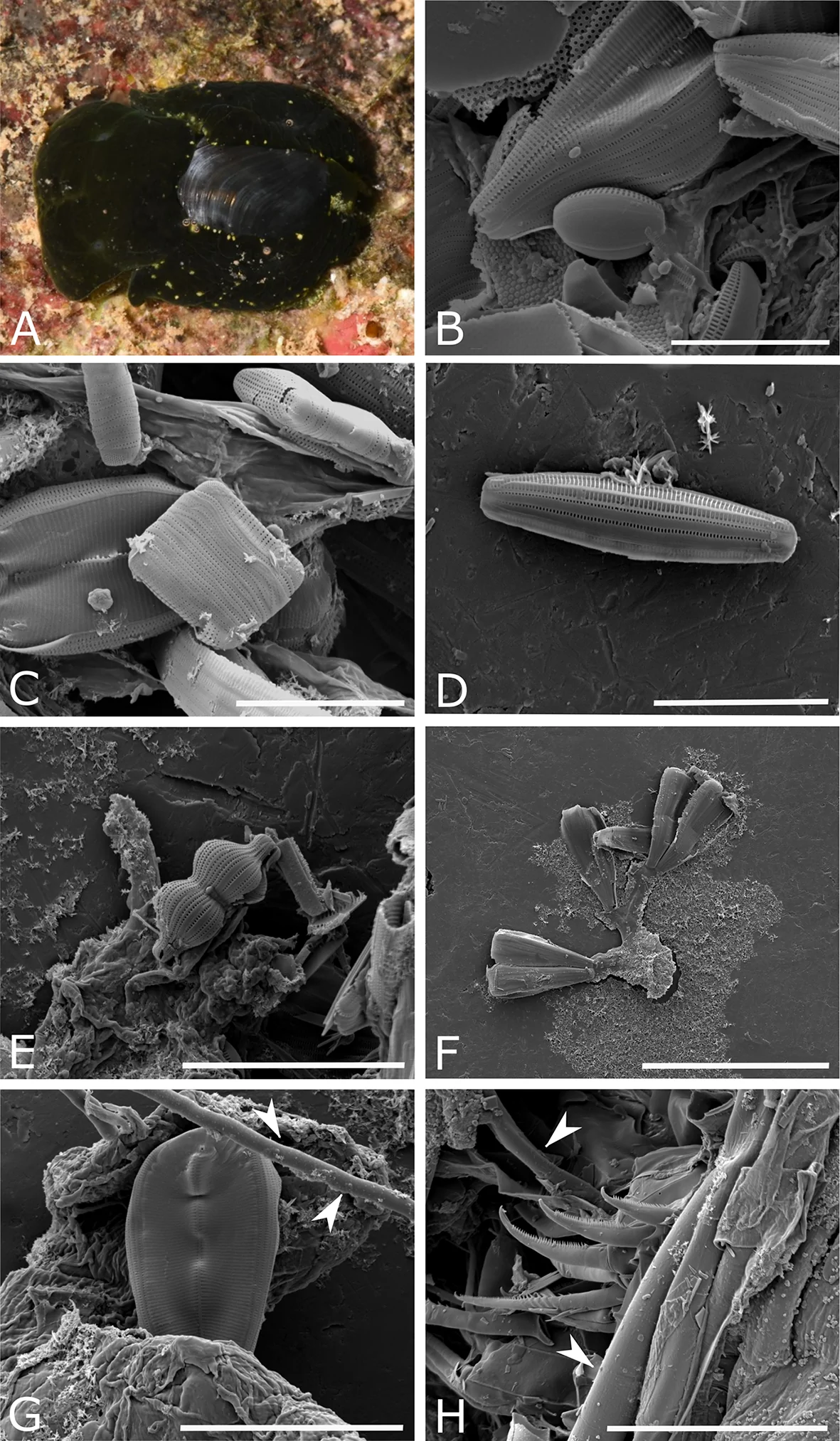
Micrographs of food items found in the gut and faeces of *Smaragdinella viridis* from Guam, Pacific Ocean. (**A**) Live animal (ZMBN 130251, animal length = 15 mm; photo by Manuel Malaquias). (**B**–**E**) Pennate diatoms. (**F**,**G**), Pennate diatom of genus *Licmophora*. (**H**) Mandibulae of branchiopod larva. Arrow heads in (**G**) and (**H**) pointing to unidentified tubular structures. Scale bars: (**B**–**D**) = 10 μm, (**E**, **G**) = 20 μm, (**F**) = 50 μm, (**H**) = 30 μm.

The SEM analysis revealed the presence of pennate diatoms, including members of the genus *Licmophora* sp. (Fig. 1B–G). In addition, part of the mandibulae of a branchiopod larva was found in a faecal pellet sample (Fig. 1H). Unidentified tubular structures possibly of plant origin were found both in the gut and faecal pellets (Fig. 1G, H).

### Eukaryote COI metabarcoding of gut and faeces

**Fig. 2:**
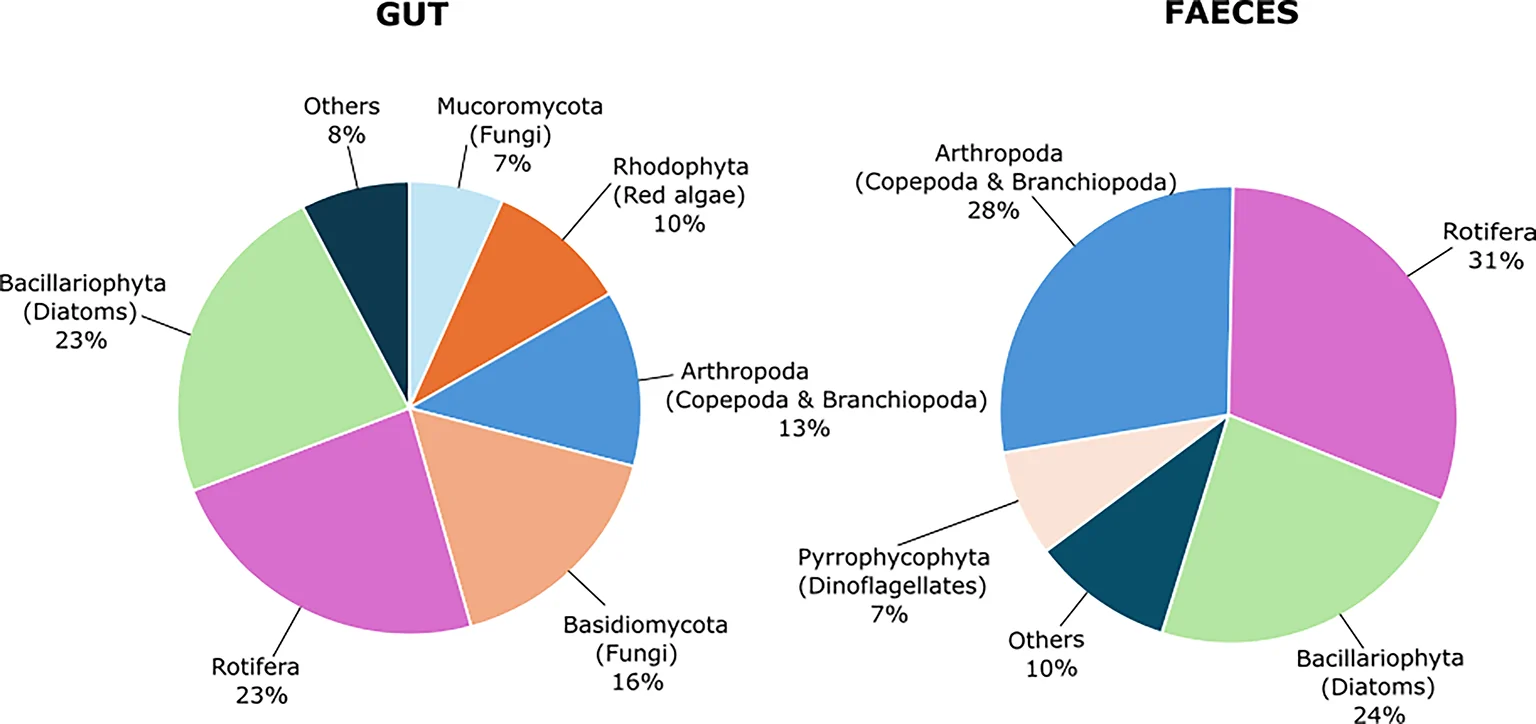
Relative abundance (%) of eukaryotes found in the gut and faeces of *Smaragdinella viridis* from Guam, Pacific Ocean.

The number of raw reads in the COI data was 105,012 and 106,667 for gut and faeces respectively, reduced to a final 41,413 (gut) and 78,747 (faeces) reads after DADA2 processing, length filtering, and chimaera detection. In the gut, the most dominant eukaryotic groups identified were Rotifera and Bacillariophyta (diatoms), each contributing 23% of the total community. These were followed by Basidiomycota (fungi) at 16%, and Arthropoda, specifically copepods and branchiopods, which together comprised 13%. Rhodophyta (red algae) accounted for 10%, while Mucoromycota (fungi) contributed 7%. The remaining 8% was grouped under “Others” which were less prevalent taxa under 5% of total number (for details see Fig. 2; Electronic supplementary material 1, 6).

**Table 1:** Diet composition of genera in the family Haminoeidae.

Genus	Diet	Habitat	References
*Aliculastrum* Pilsbry, 1896	Unknown	Sandy bottoms	Too et al.^4^
*Atys* Montfort, 1810	Unknown	Soft bottoms / coral rubble	Too et al.^4^
*Bakawan* Oskars & Malaquias, 2019	Diatoms	Algae mats / seagrass	Riek^47^; Oskars & Malaquias^12^
*Bullacta* Bergh, 1901	Diatoms	Intertidal mudflats	Malaquias^9^; Bao-Ming et al.^48^
*Confettiella* Oskars, 2024	Diatoms	Soft bottoms	Oskars^7^
*Cylichnatys* Habe, 1952	Unknown	Seagrass / mudflats	Burn^49^
*Diniatys* Iredale, 1936	Cyanobacteria	Sandy bottoms / reef flats	Gosliner et al.^18^; Too et al.^4^
*Haloa* Pilsbry, 1921	Diatoms and dinoflagellates	Algae mats / seagrass	Oskars & Malaquias^15^
*Haminella* Thiele, 1925	Unknown	River estuary in muddy bottom in brackish water / Shallow water burrowimg just under the surface of fine-grained sediments	Oskars & Malaquias^6^; Wranik & Manuel^50^
*Haminoea* W. Turton & Kingston, 1830	Diatoms	Algae mats / seagrass	See Malaquias et al.^3^ for a review
*Lamprohaminoea* Habe, 1952	Cyanobacteria, diatoms	Tidal and shallow rocky and coral reef systems	Cruz-Rivera and Paul^51^; Oskars & Malaquias^14^
*Liloa* Pilsbry, 1921	Algae or algal film	Sandy bottoms	Rudman^17^
*Papawera* Oskars & Malaquias, 2019	Filamentous algae, probably on their epiphytic diatoms	Sandy-mud flats, muddy seagrass beds, exposed rocky shores, rock platforms, tide pools and rock pools	Willan^16^; Oskars & Malaquias^6^
*Phanerophthalmus* A. Adams, 1850	Diatoms	Algae mats / seagrass	Austin et al.^5^
*Roxaniella* Monterosato, 1884	Unknown	Mud flat / on algae *Halimeda*	Too et al.^4^
*Smaragdinella* A. Adams, 1848	Rotifers, diatoms, fungi, copepods and branchiopods, rhodophytes, dinoflagellates	Hard bottoms on upper tidal zone	Current work
*Vellicolla* Oskars, Too, Rees, Mikkelsen, Willassen & Malaquias, 2019	Unknown	Algae wash + rubble + Halimeda	Oskars & Malaquias^6^
*Weinkauffia* Weinkauff, 1873	Unknown	Under mangrove roots	Collin et al.^52^

In faecal samples, Rotifera was the most abundant eukaryotic group, comprising 31% of the total community. Arthropoda followed with 28%, while Bacillariophyta remained a substantial component at 24%. Pyrrhophyta (dinoflagellates) were present at a lower proportion of 7%. The remaining 10% consisted of various low-abundance taxa, collectively grouped as “Others” (for details see Fig. 2; Electronic supplementary material 1).

### Bacterial 16S amplicon gut community

**Fig. 3:**
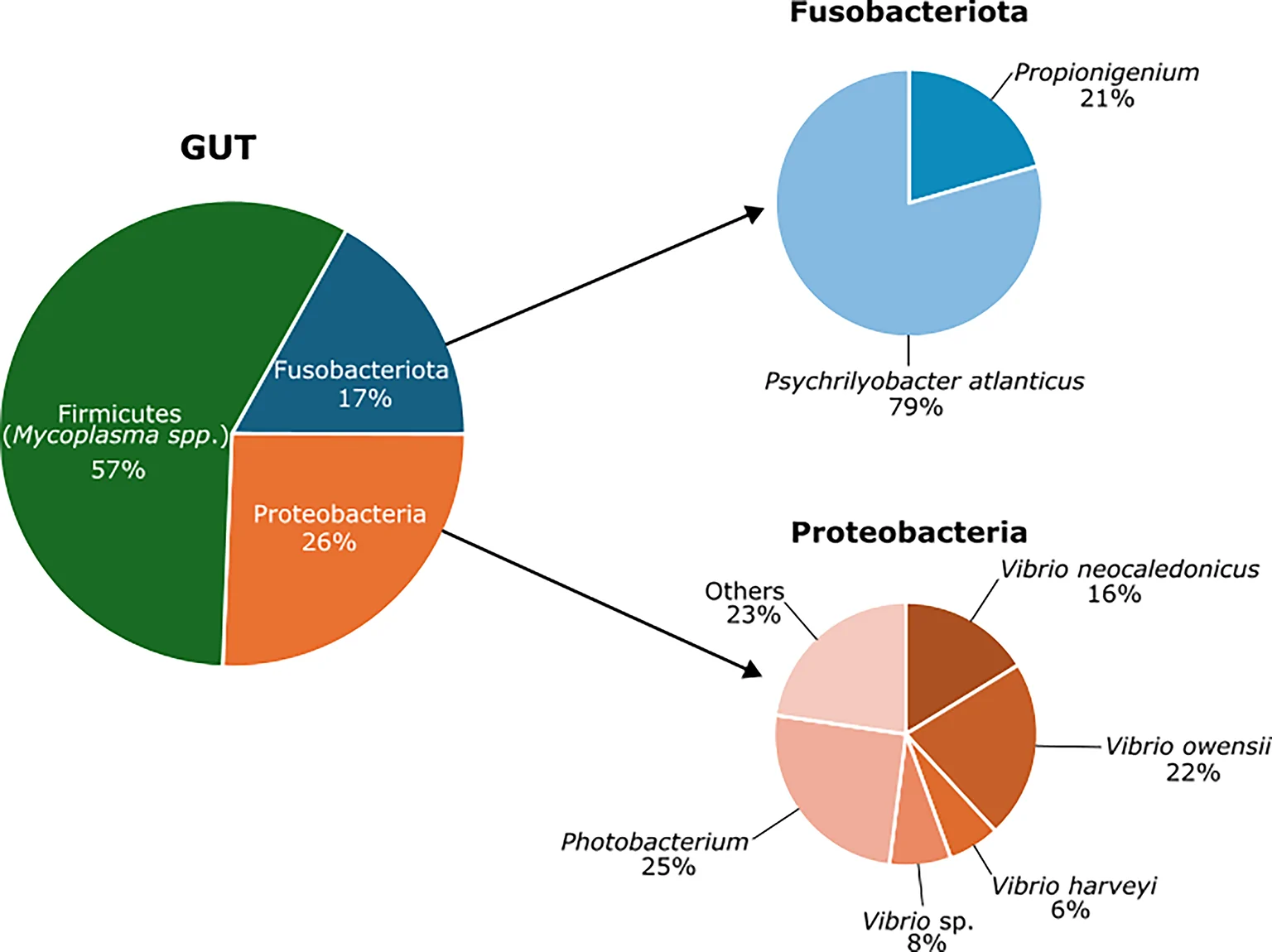
Relative abundance (%) of bacteria found in the gut of *Smaragdinella viridis* from Guam, Pacific Ocean.

The number of raw reads in the 16S data was 104,683 and 103,293 for gut and faeces respectively, reduced to a final 50,083 (gut) and 38,916 (faeces) reads after DADA2 processing, length filtering, and chimaera detection. In the gut, the bacterial community was dominated by Firmicutes, primarily the genus *Mycoplasma*, which constituted 57% of the total composition. This was followed by Proteobacteria at 26%, and Fusobacteriota at 17%. Within Fusobacteriota, *Psychrilyobacter atlanticus* was the dominant species, accounting for 79% of that phylum, while *Propionigenium* represented the remaining 21%. In contrast, the Proteobacteria community was more taxonomically diverse. *Photobacterium* was the most abundant genus, contributing 25% of the Proteobacterial fraction, followed by *Vibrio owensii* (22%), *Vibrio neocaledonicus* (16%), an unclassified *Vibrio* species (8%), and *Vibrio harveyi* (6%). The remaining 23% of the total bacterial community was classified as “Others”, comprising a range of low-abundance taxa, each contributing less than 5% (for details see Fig. 3; Electronic supplementary materials 2, 3, 6).

## Discussion

### The diet of *Smaragdinella*

Haminoeids are considered herbivore snails that feed mainly on diatoms (see Table 1 for a review). A few studies mention the ingestion of animals in *Haminoea hydatis* and *H. orbignyana*, but this was considered the result of accidental consumption during foraging on sediments and algae/seagrass^3,19,20^). The only previous study addressing diet in an unidentified species of *Smaragdinella* suggested these snails to seemingly feed on diatoms^33^, though the method used can underestimate non-diatom diversity (see Introduction).

The use of DNA metabarcoding to investigate *Smaragdinella* gut and faecal samples showed a greater diversity of food items beyond a strictly herbivorous diet. In addition to diatoms, our results revealed a substantial abundance of rotifers, fungi, and arthropods, implying that these snails are omnivorous instead of strictly herbivorous (Figs. 1 and 2).

The relatively small sampling size in this study and the use of a single mitochondrial gene marker to identify eukaryotes in the diet present some limitations to the study. Whereas the study of a few specimens from a population only offers a glimpse of the dietary regime of these snails, the COI gene marker tends to underestimate the abundance of non-metazoan organisms, meaning that relative abundances should not be taken at absolute value. The use of the nuclear ribosomal 18S gene could potentially have expanded the detectable diversity of eukaryotes, but unfortunately and despite our efforts, sequencing of the latter gene failed. Consequently, some aspects of dietary diversity and relative abundance may be underrepresented, particularly for taxa such as diatoms. Still, in the case of non-metazoan organisms the COI data did retain diatoms (Bacillariophyta) as an important part of the diet (23% in gut; 24% in faeces), but not more than rotifers, which were especially abundant (23% in gut; 31% in faeces). Pennate diatoms were similarly present in SEM images, though no rotifers were detected (Fig. 1). Other kinds of algae, including red algae (Rhodophyta) and dinoflagellates (Pyrrhophyta), fungi (Basidiomycota and Mucoromycota) were also present in small percentages and may have been assimilated incidentally. In the case of metazoans small arthropods like copepods and branchiopods also contributed substantially (13% in gut; 28% in faeces), corroborated by the presence of branchiopod remains in the SEM faecal samples.

### Is the trophic regime related with the adaption to hard substrates in *Smaragdinella*?

Herbivory is considered the plesiomorphic feeding strategy condition in the Cephalaspidea^1,8,53^, with carnivory evolving independently two or three times^1^. According to Malaquias et al.^1^, the evolutionary radiation of the Cephalaspidea does not closely conform with shell reduction or loss, lifestyle, digestive anatomy, or prey habitats. Instead, they proposed that diversification was more directly linked to dietary specialization, particularly toward two primary food sources, those of plant or animal origin. While prey structure, habitat, and anatomical traits may have had a secondary influence on the phylogeny of the Cephalaspidea, likely playing a key role in diversification within individual subclades and fostering the emergence of more specialized predator–prey interactions. 

This seems to be the case of the *Smaragdinella* snails. The genus has a unique diet among the Cephalaspidea coupled with the fact that they are the only lineage to dwell directly associated to hard substrates on the upper tidal zone. All members of the family Haminoeidae have been reported to be herbivorous feeding on diatoms (Table 1), whereas here we demonstrate that *Smaragdinella* has a more ubiquitous diet feeding on diatoms but also small arthropods, and fungi. Despite overall similarities in the digestive system of *Smaragdinella* with other members of the family Haminoeidae, this genus exhibits a chief distinctive feature in the configuration of the gizzard plates. *Smaragdinella* possesses roughly twice as many ridges (53–103 ridges^27^) compared with its closest relatives *Bakawan*, *Haloa*, *Lamprohaminoea*, and *Papawera* (7–40 ridges; with *Bakawan fusca* dwelling on mangroves as an exception displaying 50–56 ridges)^6,12–15^. The increased number of ridges likely confers an adaptive advantage by enhancing the mechanical shredding capacity of the gizzard plates, rendering them more abrasive and destructive – analogous to a kitchen bread knife – and thereby facilitating the processing of a broader spectrum of dietary items, including of both plant and animal origin with exoskeletons. This adaptation may be linked to a possible limited availability of benthic diatoms in the upper tidal zone because of harsher conditions such as wave action and air exposure causing dehydration^54^. Consequently, the higher number of ridges on the gizzard plates of *Smaragdinella* may represent a specialized morphological feeding adaptation to exploit alternative food and consequently energy sources.

Along with modifications to the digestive system, *Smaragdinella* snails evolved striking body-shape adaptations. These include a depressed body, a flattened ovoid shell that reduces body height, facilitating concealment within crevices or empty barnacle shells, and an enlarged foot that enhances adhesion to the substrate, thereby minimizing the risk of displacement by wave action. As emphasized by Bharate et al.^27^, such traits likely reflect increased fitness in high-energy shore environments and underscore the influence of ecological pressures in shaping morphological evolution within the Haminoeidae radiation.

It is plausible to hypothesise that adaptation to tidal hard bottom habitats in these snails was prompted by the acquisition of novel morphological features and by a diet shift. Even if the previous scenario seems the most parsimonious to us, we acknowledge that this putative correlation can also be explained by alternative hypotheses. For example, the acquisition of novel morphological features and diet could have been driven instead by the shift to a novel habitat, or even that all three modifications may have been acquired independently prompted by other causes.

Despite these insights, DNA metabarcoding data remain unavailable for other closely related genera occupying contrasting habitats (e.g., *Bakawan* in mangroves, *Haloa* in tropical seagrass and soft sediments, *Lamprohaminoea* in coral reefs, and *Papawera* on temperate rocky shores). Future comparative studies are essential to test the strict herbivory condition of other haminoeid snails and the hypothesis that lineage-specific dietary profiles may have contributed to habitat specialization and, ultimately, to ecological speciation within haminoeid snails.

### The gut microbiome as a potential driver of digestive efficiency

Marine invertebrates can rely on complex associations with bacteria that perform essential functions other than as prospective sources of food. Gut microbial communities can provide the host with nutrients by secreting enzymes that degrade structurally complex dietary compounds producing essential metabolites^55,56^. These symbiotic associations likely established over evolutionary timescales and contributed to shape host morphology, physiology, behaviour, and facilitated adaptation to new habitats and diet regimes^46,57–59^. For example, Duperron et al.^60^, showed that in two species of chitons with different habitat preference the gut microbiome is distinct. Utermann et al.^61^, demonstrated that gut bacteria have facilitated adaptation of the ascidian *Ciona intestinalis* to new habitats by producing toxins, improving nitrogen recycling, and increasing resistance to heavy metals. Gafarova et al.^46^, found different bacteria associated with two cryptic gastropod species of *Littorina* and Panova et al.^43^, showed that in *Littorina saxatilis* the microbiome varies between ecotypes and shore height used by the snails.

Therefore, microbiome composition not only provides information on digestive activity but also on potential evolutionary processes related to host adaptation to specific habitats^46,62^. In *Smaragdinella*, the microbial gut community seems to reflect these two conditions. Three main bacterial groups prevail in the gut of these snails, which have digestive functions providing dietary versatility and likely facilitated adaptation to rocky shores.

The Fusobacteriota, with *Propionigenium* spp. and *Psychrilyobacter atlanticus*, are anaerobe fermenters contributing to degrade simple and complex carbohydrates into short-chain fatty acids such as acetate and propionate providing readily accessible energy to the host^63,64^. Their abundance in the gut of *Smaragdinella* (17%) suggests these bacteria to be stable members of the microbiota rather than transient food-borne bacteria. Similar associations have been reported in other gastropods, highlighting the role of anaerobic fermenters in supporting host metabolism and dietary versatility^43,65,66^.

Another relevant group in the gut of our *Smaragdinella* snails was the Proteobacteria (26%), dominated by species of *Vibrio*. While *Vibrio* occurs widely in sediments and plankton^67^, and may be ingested by chance with food items, many species also degrade algal polysaccharides such as agarose, laminarin, and alginate^67,68^. Their abundance in the gut suggests a functional role aiding the digestive capacity of plant-origin dietary items.

The most abundant bacterial group was the Firmicutes (57%) dominated by species of *Mycoplasma*, which represented more than half of the microbiome community. *Mycoplasma* spp. have been reported in multiple intertidal / shallow water rocky-dwelling gastropods, including *Littorina*, *Haliotis*, and *Rapana*^43,66,69,70^, where they contribute to glycan and amino sugar degradation, nutrient absorption, and survival under nutrient-limited conditions^71^. The prevalence of *Mycoplasma* in *Smaragdinella* suggests a possible relationship that enhance host resilience and adaptive potential in the fluctuating conditions of rocky shore environments as suggested for several other marine taxa^61,62,72^. Moreover, the similarities with *Littorina*, a genus also inhabiting hard substrates on rocky shores^43^, including shared taxa such as Fusobacteriota (*Psychrilyobacter*), Firmicutes (*Mycoplasma*), and Proteobacteria (*Vibrio*) further suggest that such microbial assemblages are likely correlated with adaptation to hard-bottom intertidal habitats.

## Conclusions

Haminoeid marine snails have long been considered herbivorous, a view based primarily on traditional gut-content observations. However, the present study, applying DNA metabarcoding techniques to *Smaragdinella* for the first time, reveals a markedly different dietary pattern in these tidal, hard-bottom specialists. Contrary to previous assumptions, *Smaragdinella* haminoeids exhibits a broad omnivorous diet, consuming organisms across multiple trophic levels, including diatoms, rotifers, small arthropods, and fungi. These findings expose a dietary complexity that has gone undetected by morphological analyses alone.

The gut microbiota of *Smaragdinella* further suggests that bacteria within the digestive tract function as more than incidental food items. Instead, they appear to act as partners that enhance metabolic capacity, improve nutritional efficiency, and likely boost ecological resilience. Such microbial associations probably contribute to dietary flexibility and support survival in the dynamic and resource-variable tidal environments where these snails dwell.

Several distinctive morphological traits such as an increased number of ridges on the gizzard plates, a flattened ovoid shell, and an enlarged foot, may also facilitate the shredding of diverse food items and enhance survival in hydrodynamic tidal habitats.

Taken together, these results suggest that the ecological success of *Smaragdinella* within this unique habitat, unmatched among other members of the order Cephalaspidea, has likely been shaped by the combined evolution of specialized morphology, dietary versatility, and perhaps a functionally significant gut microbiome.

## Materials and methods

### Taxon sampling, gut content, and faeces isolation

Sixteen specimens of *Smaragdinella viridis* were collected in Pago Bay, Guam, Mariana Islands on the 30th of November 2023 on the upper tidal zone of rocky outcrops during low tide. Specimens were measured and photographed alive in the field and laboratory using a digital DSLR camera equipped with a macro lens. In the laboratory, the specimens were transferred to a tray with sea water and approximately 1 cm^3^ of faecal pellets was randomly collected with the aid of a Pasteur pipette and preserved inside an Eppendorf tube with 96% ethanol. Afterwards, specimens were frozen in sea water overnight to ensure they died with their bodies fully extended, defrosted the following day, and preserved in 96% ethanol.

Six specimens were dissected by dorsal incision for their digestive tract. The oesophagus, gizzard, stomach, and intestine were separated, opened, and the gut contents were carefully removed. Gut contents from four specimens were pooled into a single Eppendorf tube for metabarcoding analysis to obtain sufficient DNA, while contents from the remaining two specimens were combined in another Eppendorf tube for scanning electron microscopy (SEM). Both samples were preserved in 96% ethanol. Faecal pellets were divided into two Eppendorf tubes containing 96% ethanol, one for metabarcoding and the other for SEM analysis.

### Scanning electron microscopy analysis of gut content and faeces

The Eppendorf tubes containing the gut contents and faecal pellets were homogenized using a vortex mixer (VWR Lab Dancer S040) for 10–20 s. With a Pasteur pipette 3–4 drops of the mixture were transferred onto metallic stubs covered with carbon sticky tabs. Eight stubs were prepared and let to dry on air and afterwards coated with gold-palladium. Examination and imaging of gut contents was carried out with a FEI Quanta 450 scanning electron microscope.

### Molecular metabarcoding analyses

DNA extractions were done with the BayBiopure kit. Quality of the DNA was checked with gel electrophoresis and nanodrop. We targeted three genes, the mitochondrial COI and nuclear 18S rRNA gene to profile eukaryotes, and the mitochondrial 16S rRNA gene to profile bacterial communities. DNA quality was not sufficient for 18S rDNA, which hindered our efforts to sequence this gene. The COI gene was amplified using the forward primer mlCOIintF-XT 5’- GGWACWRGWTGRACWITITAYCCYCC − 3’^73^, and the reverse primer jgHCO2198 5’- TAIACYTCIGGRTGICCRAARAAYCA − 3’^74^. The 16S rRNA gene was amplified using Eubacteria forward primer 314 F 5’- CCTACGGGNGGCWGCAG-3’ and reverse primer 805R 5’- GACTACHVGGGTATCTAATCC-3’^75^. PCR for COI and 16S reactions were carried out with 15 µL of Phusion High - Fidelity PCR Master Mix; 0.2 µM of forward and reverse primers, and about 10 ng template DNA. Thermal cycling consisted of initial denaturation at 98℃ for 1 min, followed by 30 cycles of denaturation at 98℃ for 10 s, annealing at 50℃ for 30 s, and elongation at 72℃ for 30 s and 72℃ for 5 min. The PCR products were purified using magnetic beads and diluted to equimolar concentration. Individual PCR products were assessed on an agarose gel for fragmentation of DNA. Libraries for COI and 16S were prepared using the NEBNext^®^ Ultra™ IIDNA Library Prep Kit (Cat No. E7645) following the manufacturer’s protocol. The library was inspected with Qubit and real-time PCR for quantification and bioanalyzer for size distribution detection. Quantified libraries were pooled and sequenced, according to effective library concentration and data amount required and sequenced using Illumina Sequencing PE250 at Novogene, Germany.

### Data processing and bioinformatics analysis

Raw sequence data was treated with Cutadapt^76^, to remove primer sequences. Quality filtering, trimming, denoising, dereplication and *de-novo* chimera detection was done using DADA2^77^, with length filtering excluding sequences not within length range 307–320 base pairs (COI) and 300–450 base pairs (16S), respectively. To remove cross contaminants between samples, an in-house analogue of UNCROSS2^78^ was used. Taxonomic assignment of Amplicon Sequence Variant (ASV) tables was done using VSEARCH 2.29 syntax classification (16S)^79^, and for COI sequences with BOLDigger3^80^, using the BOLD–The Barcode of Life Data Systems V5 (https://boldsystems.org/).

Once the ASV table was generated, we removed sequences of the host taxon, Chordata taxa, incomplete taxonomy, and reads with no match. Then, we calculated the relative abundance in percentage of each taxon present in the gut and faecal samples for eukaryotes and bacteria by dividing each read count by the total number of reads and multiplying by 100. Only taxa with a relative abundance greater than 5% were labelled individually in the pie chart; those below this threshold were grouped under “Others”. This conservative abundance threshold was done to reduce the number of potential contaminant sequences in the data (see Electronic supplementary material 1–3, 6).

## Supplementary Information

Below is the link to the electronic supplementary material.

Supplementary Material 1

Supplementary Material 2

Supplementary Material 3

Supplementary Material 4

Supplementary Material 5

Supplementary Material 6

## Data Availability

The sequencing data in this study are available in GenBank under SRA accession: PRJNA1188506.
