# Supplementary material for: Molecular metabarcoding reveals an omnivorous diet in Smaragdinella haminoeid snails and sheds light on their unique evolution
Source: Sci Rep. 2026 Jul 9;16:25711. doi: 10.1038/s41598-026-57881-9 (PMC13482812; doi:10.1038/s41598-026-57881-9)
Supplement: Supplementary file 1 [file 41598_2026_57881_MOESM1_ESM.docx]

Supplementary Material 1: Details on eukaryotes found in Gut and Faeces

| COI | **Gut** (Reads) | **%** | **Faeces** (Reads) | **%** |
| --- | --- | --- | --- | --- |
| Annelida | 0 | 0.00 | 27 | 0.34 |
| Chlorophyta | 0 | 0.00 | 11 | 0.14 |
| Cryptophyta | 0 | 0.00 | 15 | 0.19 |
| Haptophyta | 0 | 0.00 | 221 | 2.75 |
| Heterokontophyta | 0 | 0.00 | 27 | 0.34 |
| Cnidaria | 2 | 0.40 | 20 | 0.25 |
| Ochrophyta | 3 | 0.60 | 110 | 1.37 |
| Porifera | 5 | 1.00 | 0 | 0.00 |
| Ascomycota | 8 | 1.60 | 2 | 0.02 |
| Pyrrophycophyta | 20 | 3.99 | 605 | 7.52 |
| Mucoromycota | 33 | 6.59 | 51 | 0.63 |
| Rhodophyta | 50 | 9.98 | 318 | 3.95 |
| Arthropoda | 63 | 12.57 | 2266 | 28.15 |
| Basidiomycota | 83 | 16.57 | 0 | 0.00 |
| Bacillariophyta | 117 | 23.35 | 1909 | 23.71 |
| Rotifera | 117 | 23.35 | 2468 | 30.66 |
| Total | **501** | **100%** | **8050** | **100%** |
