# Supplementary material for: Molecular metabarcoding reveals an omnivorous diet in Smaragdinella haminoeid snails and sheds light on their unique evolution
Source: Sci Rep. 2026 Jul 9;16:25711. doi: 10.1038/s41598-026-57881-9 (PMC13482812; doi:10.1038/s41598-026-57881-9)
Supplement: Supplementary file 2 [file 41598_2026_57881_MOESM2_ESM.docx]

Supplementary Material 2: Details on bacteria found in Gut

| 16S | **Gut** (Reads) | **%** |
| --- | --- | --- |
| Chloroflexi | 0 | 0.00 |
| Deinococcota | 0 | 0.00 |
| Entotheonellaeota | 0 | 0.00 |
| Dependentiae | 2 | 0.00 |
| Myxococcota | 2 | 0.00 |
| Campilobacterota | 3 | 0.01 |
| Actinobacteriota | 8 | 0.02 |
| SAR324_clade (Marine_group_B) | 9 | 0.02 |
| Desulfobacterota | 10 | 0.02 |
| Acidobacteriota | 12 | 0.02 |
| Spirochaetota | 25 | 0.05 |
| Verrucomicrobiota | 57 | 0.11 |
| Cyanobacteria | 67 | 0.13 |
| Bdellovibrionota | 86 | 0.17 |
| Bacteroidota | 272 | 0.54 |
| Planctomycetota | 450 | 0.90 |
| Fusobacteriota | 8294 | 16.56 |
| Proteobacteria | 12557 | 25.07 |
| Firmicutes | 28229 | 56.36 |
| Total | **50083** | **100%** |
