# Supplementary material for: Molecular metabarcoding reveals an omnivorous diet in Smaragdinella haminoeid snails and sheds light on their unique evolution
Source: Sci Rep. 2026 Jul 9;16:25711. doi: 10.1038/s41598-026-57881-9 (PMC13482812; doi:10.1038/s41598-026-57881-9)
Supplement: Supplementary file 3 [file 41598_2026_57881_MOESM3_ESM.docx]

Supplementary Material 3: Species level breakdown of bacteria in Gut

| **Break down of 16S** |  | **Gut (Reads)** | **%** |
| --- | --- | --- | --- |
| **Proteobacteria** |  | 12557 |  |
| Proteobacteria | *Vibrio neocaledonicus* | 2038 | 16.23 |
| Proteobacteria | *Vibrio owensii* | 2750 | 21.90 |
| Proteobacteria | *Vibrio harveyi* | 796 | 6.34 |
| Proteobacteria | *Vibrio* sp. | 949 | 7.56 |
| Proteobacteria | *Photobacterium* | 3175 | 25.28 |
| Others | Others | 2849 | 22.69 |
| **Fusobacteriota** |  | 8294 |  |
| Fusobacteriota | *Propionigenium* | 1691 | 20.39 |
| Fusobacteriota | *Psychrilyobacter atlanticus* | 6547 | 78.94 |
| Others |  | 56 | 0.68 |
| **Firmicutes** |  | 28229 |  |
|  | *Mycoplasma* | 28207 | 99.92 |
| Others |  | 22 | 0.08 |
