# Supplementary material for: Molecular metabarcoding reveals an omnivorous diet in Smaragdinella haminoeid snails and sheds light on their unique evolution
Source: Sci Rep. 2026 Jul 9;16:25711. doi: 10.1038/s41598-026-57881-9 (PMC13482812; doi:10.1038/s41598-026-57881-9)
Supplement: Supplementary file 4 [file 41598_2026_57881_MOESM6_ESM.docx]

Eukaryotes represented by a relative abundance < 5% found in the gut and faeces of *Smaragdinella viridis* from Guam, Pacific Ocean. Values on y-axis are percentages.

Bacteria represented by a relative abundance < 5% found in the gut and faeces of *Smaragdinella viridis* from Guam, Pacific Ocean. Values on y-axis are percentages.
